# Cell death by the quinoxaline dioxide DCQ in human colon cancer cells is enhanced under hypoxia and is independent of p53 and p21

**DOI:** 10.1186/1748-717X-5-107

**Published:** 2010-11-15

**Authors:** Mona El-Khatib, Fady Geara, Makhluf J Haddadin, Hala Gali-Muhtasib

**Affiliations:** 1 Department of Biology, American University of Beirut, Beirut, Lebanon; 2 Department of Radiation Oncology, American University of Beirut, Beirut, Lebanon; 3 Department of Chemistry, American University of Beirut, Beirut, Lebanon

## Abstract

**Introduction:**

We have shown that the radio sensitizer DCQ enhances sensitivity of HCT116 human colon cancer cells to hypoxia. However, it is not known whether the p53 or p21 genes influence cellular response to DCQ. In this study, we used HCT116 that are either wildtype for p53 and p21, null for p53 or null for p21 to understand the role of these genes in DCQ toxicity.

**Methods:**

HCT116 cells were exposed to DCQ and incubated under normoxia or hypoxia and the viability, colony forming ability, DNA damage and apoptotic responses of these cells was determined, in addition to the modulation of HIF-1α and of p53, p21, caspase-2, and of the ataxia telangiectasia mutated (ATM) target PIDD-C.

**Results:**

DCQ decreased colony forming ability and viability of all HCT116 cells to a greater extent under hypoxia than normoxia and the p21^-/-^cell line was most sensitive. Cells had different HIF-1α responses to hypoxia and/or drug treatment. In p53^+/+^, DCQ significantly inhibited the hypoxia-induced increases in HIF-1α protein, in contrast to the absence of a significant HIF-1α increase or modulation by DCQ in p21^-/- ^cells. In p53^-/- ^cells, 10 μM DCQ significantly reduced HIF-1α expression, especially under hypoxia, despite the constitutive expression of this protein in control cells. Higher DCQ doses induced PreG_1_-phase increase and apoptosis, however, lower doses caused mitotic catastrophe. In p53^+/+ ^cells, apoptosis correlated with the increased expression of the pro-apoptotic caspase-2 and inhibition of the pro-survival protein PIDD-C. Exposure of p53^+/+ ^cells to DCQ induced single strand breaks and triggered the activation of the nuclear kinase ATM by phosphorylation at Ser-1981 in all cell cycle phases. On the other hand, no drug toxicity to normal FHs74 Int human intestinal cell line was observed.

**Conclusions:**

Collectively, our findings indicate that DCQ reduces the colony survival of HCT116 and induces apoptosis even in cells that are null for p53 or p21, which makes it a molecule of clinical significance, since many resistant colon tumors harbor mutations in p53.

## Introduction

Hypoxia develops due to the inadequate vascularization during early tumor development and is believed to be the major factor causing tumor resistance to radiotherapy and chemotherapy [1]. Numerous gene products, which are activated under hypoxia, are involved in tumor metastasis and neoangiogenesis. On the other hand, hypoxic cells contain high levels of bioreductive enzymes and thus represent a therapeutic target if directly targeted by hypoxia-activated drugs [2].

Quinoxaline 1,4-dioxides (QdNOs) are the prototype for current heterocyclic N-oxide anticancer agents such as 3-amino-1,2,4-benzotriazine 1,4-dioxide (Tirapazamine-TPZ). Among four QdNOs tested, we found DCQ (2-benzoyl-3-phenyl 6,7-dichloroquinoxaline 1,4-dioxide) to be the most effective hypoxic cytotoxin [3-6]. Although DCQ is not a benzotriazine 1,4-dioxide like TPZ, it resembles TPZ in that these two compounds are electron-poor by virtue of the formal positive charges held by the two nitrogens of the N-O functions in each of them. In fact, DCQ is believed to be more electron-poor than TPZ because it has more electron attracting substituents: the 2-benzoyl group and the 6,7-dichloro substituents. These substituents render the quinoxaline 1,4-dioxide moiety more receptive to an electron from a donor. Furthermore, and in analogy with the mechanism of action of TPZ [7], the radical that results from addition of an electron to C2 of DCQ is more stable, by resonance, and therefore longer lasting and more damaging to DNA than the radical resulting from the addition of an electron to TPZ.

DCQ was shown by our group to reduce cell growth in T-84 human colon cancer cells, and in SP-1 keratinocyte cell line, under both normoxia and hypoxia; however, drug toxicity was greater in cells exposed to hypoxia [3]. DCQ was found to decrease the expression levels of the hypoxia inducible factor (HIF-1α) mRNA and protein in the human colon carcinoma cell line T-84, and in EMT6 mouse mammary carcinoma cells and Lewis Lung Carcinoma (LLC) cells [4,8]. We also showed that DCQ inhibited cell proliferation and induced apoptosis in colon T-84 cancer cell lines under normoxia via the inhibition of the extracellular signal regulated kinase (ERK) phosphorylation and reduction in Bcl-2α protein [9]. While in adult T-cell leukemia, DCQ reduced cell proliferation by decreasing Tumor Growth Factor (TGF)-α, a key mediator of growth stimulation with mitogenic effects, and by increasing the mRNA and protein expression levels of the proapoptotic TGF-β1 [6]. When studying the efficacy of DCQ as a normoxic radiosensitizer, clonogenic survival assays in LLC and EMT6 cell lines revealed an enhancement of the radiation effect [8,10]. *In vivo*, DCQ in combination with radiation delayed the growth of LLC tumors injected in C57BL6 mice, reduced the mean tumor volume by 80% and inhibited tumor angiogenesis [8]. In a recent study, DCQ was found to induce single strand breaks (SSB) in DNA of DLD-1 human colon cancer cells, and both SSB and double strand breaks (DSB) in EMT6 cells [5,11].

DNA damage, in particular DSBs, imposes a critical threat to the survival of cells if left unrepaired [12]. At very early stages of the DNA damage response, cells activate the DNA damage checkpoint ATM, a member of phosphoinositide 3 kinase-related kinase (PIKK) which is involved in DNA repair [13]. ATM activation, in turn, leads to the phosphorylation of p53, thereby blocking its interactions with MDM2, and causing p53 stabilization. This, in turn, stimulates the expression of the cyclin-dependent kinase (CDKs) inhibitor p21. Through its negative effects on various CDKs, p21 inhibits G1/S and G2/M transitions. Thus, increased p53 levels due to the ATM-p53-p21 pathway activation lead to cell-cycle arrest, repair, and cell death [14]. Tumor cells that harbor defective p53 have no such checkpoint mechanisms, which favor their clonal outgrowth. The activation of ATM also leads to the activation of PIDD (p53-induced protein with a death domain), an important target gene in a signaling pathway initiated by p53. The tumor suppressor protein p53 has been also found to be activated in response to cellular stress, chemotherapeutic drugs and hypoxia [15].

If DNA damage is severe, the initiator caspase-2 is activated. This caspase possesses a caspase recruitment domain (CARD) that allows it to interact with PIDD. Caspase-2 activation can be initiated in the PIDDosome, the assembly of which is mediated by PIDD autoprocessing to generate a PIDD-CC fragment necessary for caspase-2 activation [16]. A recent study has demonstrated that p53 controls the expression of PIDD that, in turns recruits procaspase-2 by interaction with its prodomain [16]. The resulting complex activates caspase-2 without interdomain cleavage of caspase-2 [16]. The activation of caspase-2 within the PIDDosome complex results in cytochrome c release and the activation of other caspases which are involved in the mitochondria-mediated apoptotic pathway [17]. Caspase-2 activation has been shown to be involved in metaphase-associated mitotic catastrophe [17], which is characterized by multinucleated giant cells with nuclear envelopes forming around individual clusters of mis-segregated uncondensed chromosomes [17].

This project aimed to investigate the cytotoxicity of DCQ in HCT116 human colorectal cancer cell lines that are either wildtype for p53 and p21, null for p53, or null for p21 to determine the role of these genes in cellular response to DCQ. Since DCQ has been previously shown to exhibit enhanced toxicity in hypoxic tumor cells, its activity was determined in HCT116 cells exposed to either normoxia or hypoxia. We also investigated if DCQ causes apoptosis, induces SSB and activates the ATM repair pathway in human colon cancer cells.

## Methods

### Chemicals

Propidium iodide (PI), YOYO-1 dye, fluorescein isothiocyanate (FITC) goat anti-mouse IgG (H+L), and 5-(and-6)-chloromethyl-2',7'-dichlordihydrofluorescein diacetate, acetyl ester (CM-H_2_DCFDA) were purchased from Molecular Probes (Eugene, Oregon, US). RNase A, and dimethylsulfoxide (DMSO) were obtained from Sigma Chemical Company (St. Louis, Missouri, US). Protease Inhibitor was from Roche Applied Science (Penzberg, Germany). DCQ was synthesized from 5,6-dichlorobenzofurazan oxide and dibenzoylmethane by the Beirut Reaction [18].

### Cell culture and treatments

FHs74Int normal human intestinal cells were cultured in Hybri-Care medium supplemented with 30 ng/ml epidermal growth factor. HCT116 (p53^+/+^) human colon cancer cells were maintained in RPMI 1640 with 25 mM Hepes and L-Glutamine. HCT116 (p53^-/-^) and HCT116 (p21^-/-^) cells were grown in Dulbecco's Modified Eagle Medium (DMEM) supplemented with sodium pyruvate and 4500 mg/l glucose. All media were supplemented with 1% Penicillin-Streptomycin (100 U/ml) and 10% heat-inactivated FBS. All cells were obtained from ATCC and maintained in a humidified atmosphere of 5% CO_2 _and 95% air. 10 mg of DCQ was dissolved in 1 ml of DMSO and stored in a brown eppendorf at 4°C and then diluted in media to attain the drug concentrations of up to 10 μM. For hypoxia exposure, cells were placed in a tightly sealed anaerobic gas chamber, Bactron III (SHEL LAB, UK) at 37°C and oxygen level < 2%. The desired oxygen level was monitored by an Ohmeda Oxymeter (Datex-Ohmeda, Louisville CO, USA) and maintained by pumping a gas mixture composed of 1% O_2_, 5% CO_2_, and 94% N_2_. After 6 hr of hypoxia exposure, cells were replated with drug-free media and incubated under normal oxygen for clonogenic survival assays.

### Viability and clonogenic survival

For viability assays, HCT116 or FHs74Int (1.2 × 10^5 ^cells/ml) were cultured in 96-well plates and treated with drugs 24 hrs after plating. Antineoplastic effects were studied 6 hrs or 24 hrs after treatment by the non-radioactive cell proliferation kit (Promega Corporation, Madison, USA), an MTT-based method which measures the ability of metabolically active cells to convert tetrazolium salt into a formazan product and its absorbance is recorded at 570 nm [19]. For clonogenic survival studies, cells were treated with DCQ for 12 hr under normoxia or hypoxia. Then they were trypsinized, replated at low densities (300-5000 cells) in T-25 flasks, and left for 8-14 days in the incubator. Subsequently, cells were washed with PBS, and stained with 1 ml of aqueous 0.5% solution of crystal violet. Colonies having more than 50 cells were counted. The plating efficiency (PE), defined as the ability of control cells to survive and grow into colonies, was calculated as: PE = colonies counted in control/plating density of control. Surviving fraction (SF) for each treatment was calculated as: SF = colonies counted/[cells plated × (PE/100)]. The SF value of each treatment was then plotted.

### Flow cytometric analysis

Cells were plated in 60-mm dishes (1.2 × 10^5 ^cells/ml), treated with different DCQ concentrations at 50% confluency, and incubated for 6 hrs under either normoxia or hypoxia, then harvested and fixed in 70% ethanol. Supernatants containing the dead cells were collected and attached live cells were harvested by 2× trypsin and added to the supernatant. Flow cytometry analysis of Propidium Iodide-stained DNA was done as described previously [19]. Cell Quest program was used to determine the percentages of cells in various cell cycle phases. Pre-G_1 _cells with DNA content < 2n represent apoptotic or necrotic cells.

### Hoechst staining

Cells were plated in 6-well plates at 1.2 × 10^5 ^and treated at 50% confluency with DCQ (2.5 or 5 μM) for 6 hrs under normoxia or hypoxia. The drug was then removed, and cells were washed with 1× PBS and fixed using 70% ethanol for 24 hrs. Next day, cells were placed in wet chambers to prevent dehydration, a stock of Hoechst stain (1:100) was prepared and 100 μl of the 100× diluted Hoechst stain (from the stock) was added to each slide and incubated for 10 min. A drop of fluorosave (antifade) was added on the slides which were covered with coverslips and kept in the dark at 4°C.

### Annexin V

Cells were collected along with the supernatant and centrifuged at 1500 rpm for 10 min, 4°C. The pellet was washed with PBS and centrifuged at 1500 rpm for 10 min, 4°C. The pellet was resuspended in 100 μl Annexin-V-Fluos labeling solution (20 μl annexin reagent and 20 μl PI (50 μg/ml) in 1000 μl incubation buffer pH 7.4 (10 mM Hepes/NaOH, 140 mM NaCl, 5 mM CaCl_2_). The samples were incubated for 15 min at room temperature and 0.4 ml incubation buffer was added. The cellular fluorescence was then measured by flow cytometry using a Fluorescence Activated Cell Sorter (FACS) flow cytometer (Becton Dickinson, Research Triangle, NC).

### Western blot

Cellular protein extracts were prepared and proteins were quantified as described previously [19]. 50 μg of whole cell lysate was separated by SDS-PAGE (12% gels) and transferred to PVDF membranes (Amersham Pharmacia Biotech, Buckinghamshire, UK) in cold transfer buffer at 30 Volts overnight. The membranes were probed with the primary antibodies: p21 ((C-19)-G), p53 (DO-1), caspase-2 (all from Santa Cruz, California), ATM kinase phosphoser1981 antibodies (Chemicon International, California), PIDD (Alexis Biochemicals, Playmouth, USA), HIF-1α (Novus Biologicals, Littleton, USA), followed by horseradish peroxidase-conjugated anti-mouse, anti-rabbit, or anti-goat IgG-HRP (all from Santa-Cruz, California, US). GAPDH (Biogenesis, Poole, UK) was used to ensure equal protein loading. The immunoreactive bands were visualized on X-ray film with chemiluminescent substrate (Santa-Cruz). To quantify protein bands, densitometry was done using LabWorks 4.0 software. Bands were quantitated with ImageQuant software and the Molecular Dynamics Storm 860 System (Molecular Dynamics, Sunnyvale, CA).

### Alkaline comet

The alkaline comet assay used is a modification of the method developed by Singh [20]. This method which was described by us previously [11] detects the frequency of SSBs and alkaline-labile lesions in DNA. Images of a minimum of 50 cells per treatment were analyzed using the CometScore™software. Percentage of DNA in the tail region, and tail moment (% DNA in tail × by tail length (μm)) were used as parameters to assess DNA damage.

### p-ATM immunocytochemistry

Ser-1981-phosphorylated ATM was detected immunocytochemically by multiparameter cytometry with respect to the cell cycle phases, using the method developed by Huang and Darzynkiewicz [21]. Cells were collected by trypsinization, centrifuged, washed with PBS, and fixed with ice-cold 70% ethanol for a minimum of 2 hr at -20°C. Ethanol was discarded by centrifugation at a speed of 10000 rpm for 5 min, and the pellets were washed with BSA-T-PBS containing 1% BSA and 0.2% Triton X-100 dissolved in PBS. The pellets were blocked in BSA-T-PBS for 5 min at room temperature. After removal of the 1% BSA solution by centrifugation, the cells were incubated with the primary antibody Ser-1981-p-ATM at a dilution of 1:100 overnight at 4°C. The cells were washed twice with BSA-T-PBS, and the pellets were then incubated in the dark with fluorescein isothiocyanate (FITC)-conjugated secondary anti-mouse antibody (1:30) for 1 hr at room temperature. BSA-T-PBS (5 ml) was added to the cell suspension and kept for 2 min before centrifugation at 12000 rpm for 4 min. Finally, the cells were counterstained with PI (5 μg/ml) solution containing RNase A (0.1 mg/ml) for 30 min at room temperature in the dark. Both the fluorescence of PI and FITC of 10^4 ^cells/treatment were measured using the FACS cytometer, and analyzed using Cell Quest.

## Results

### DCQ decreases colon cancer cell growth to a greater extent under hypoxia

**Figure 1:**
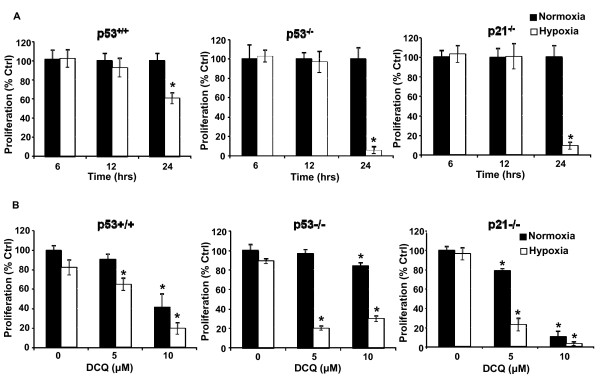
**DCQ reduces the viability of HCT116 cells more so under hypoxia than normoxia**. (**A**) The effect of hypoxia on HCT116 (p53^+/+^, p53^-/-^, p21^-/-^) cell viability after 6, 12, or 24 hrs of exposure to 1% O_2_. Cells were plated in 96 well plates at 1.2 × 10^5 ^cells/ml and treated at 50% confluency. Viability was determined using Cell Titer 96 non-radioactive proliferation assay. **(B) **Dose-dependent decrease in the viability of cells exposed to DCQ for 6 hrs and cultured under normoxia or hypoxia. Values are averages ± SD of two independent experiments each done in triplicates; (*) indicates p < 0.05 (one way ANOVA). ■ Normoxia □ Hypoxia. The experiment was repeated three times each in quadruplicates.

We have previously shown that DCQ is a hypoxic cytotoxic compound that induces apoptosis in several murine and human cancer cell lines [4,5,8]. This is our first attempt to understand the role of p53 and p21 in drug efficacy using colon cancer cells that are wildtype or null for p53 and p21. Before studying DCQ efficacy under hypoxia, we determined the sensitivity of the colon cancer cell lines to hypoxia. HCT116 (p53^+/+^, p53^-/-^, and p21^-/-^) cells were exposed to 1% O_2 _for 6, 12 or 24 hrs, after which cell viability was determined by the MTT-based Cell Titer Promega assay (Figure 1A). Although up to 12 hrs of hypoxia had no effect on viability, 24 hrs reduced it by 50% in p53^+/+ ^cells and by more than 80% in p53^-/- ^and p21^-/- ^cells (Figure 1A). Therefore, all further experiments were conducted by exposing cells to 6 or 12 hrs hypoxia. To determine the antineoplastic effects of DCQ, cells were treated with 5 or 10 μM DCQ for 6 hrs and cultured under normoxia or hypoxia. These doses are in the IC_50 _range for p53^+/+ ^cells [5]. As shown in Figure 1B, DCQ inhibited the viability of all three HCT116 cell lines in a dose-dependent fashion, and this inhibition was 2-5 fold higher under hypoxia than normoxia. p21^-/- ^cells appeared to be more sensitive to DCQ at 10 μM than the other two cell lines (Figure 1B).

**Figure 2:**
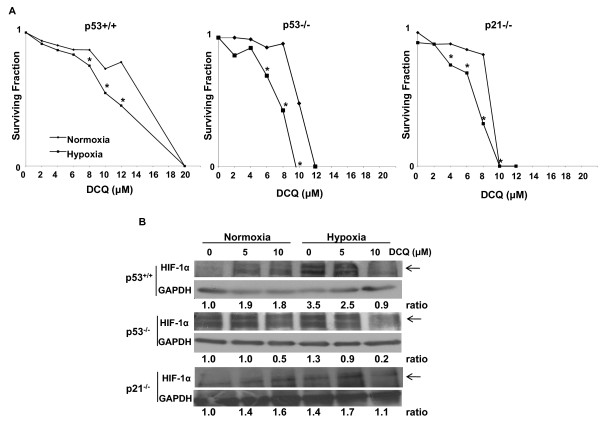
**DCQ reduces the clonogenic survival of HCT116 cells more so under hypoxia than normoxia**. **(A) **Clonogenic survival of DCQ-treated cells exposed to normoxic or hypoxic conditions. At 50% confluency, cells were treated for 12 hrs with different DCQ concentrations in normoxia or hypoxia, after which they were replated at low densities and colonies (more than 50 cells) were stained and counted after 10-14 days in culture. Surviving fractions were calculated as mentioned in "Methods". (*) indicates p < 0.05 (one way ANOVA). **(B) **Effect of DCQ on HIF-1α protein expression. Cells were plated in 100 mm dishes and treated for 6 hrs with DCQ while in normoxia or hypoxia. Whole cell lysates were immunoblotted for HIF-1α. GAPDH was used to ensure equal loading. Relative densitometry values are presented at the bottom of the blots. All ratios were normalized to GAPDH and calculated relative to the control cells cultured under oxia. The experiment was repeated three times each in triplicates.

Further studies to confirm the higher drug activity under the reducing conditions of a hypoxic environment involved carrying out clonogenic survival assays. Cells were treated with DCQ at concentrations ranging from 1-20 μM for 6 hrs (data not shown) or 12 hrs, and exposed to normoxia or hypoxia, after which cells were re-plated at low density and incubated for 8-14 days. Colonies having more than 50 cells were counted and surviving fractions were plotted (Figure 2A). DCQ decreased the colony forming ability in a dose-dependent fashion for all three cell lines under both normoxic and hypoxic conditions; however, the effect was more pronounced under hypoxia and in p21^-/- ^cells. In accordance with the MTT results, the clonogenic survival experiment indicated p21^-/- ^as drug sensitive and p53^+/+ ^as relatively more resistant.

### DCQ modulates HIF-1α protein differently in the three cell lines

To determine whether differences in drug efficacy was related to the modulation of HIF-1 α, the three cell lines were exposed to DCQ (6 hr incubation with 5 μM or 10 μM) under normoxia or hypoxia and the expression of HIF-1α protein was determined (Figure 2B). The level of HIF-1α in hypoxic tumors is known to increase to regulate metabolic adaptation to oxygen deprivation and angiogenesis [22-24]. This renders cancer cells better able to survive in the harsh hypoxic conditions [25]. Therefore, inhibiting HIF-1α-mediated signaling is important for enhancing anticancer drug efficacy. Differences in HIF-1α responses to hypoxia exposure and/or drug treatment were observed in the three cell lines. In p53^+/+ ^cells, the HIF-1α protein levels increased by 3.5 fold when cells were exposed to hypoxia, and this increase was significantly inhibited by 10 μM DCQ (Figure 2B). This is in contrast to the observed increase in HIF-1α in response to 5 μM or 10 μM DCQ under normoxia in this cell line. In p53^-/- ^cells, however, HIF-1α protein was constitutively expressed under normoxia and hypoxia, yet 10 μM DCQ reduced its expression especially under hypoxia (Figure 2B). It is interesting to note that hypoxia selects for tumors that are mutant for p53 [26]. In p21^-/- ^cell, although DCQ altered the protein level pattern of HIF-1α, no dose-dependent increase in HIF-1α was observed (Figure 2B).

### Low DCQ doses induce mitotic catastrophe while high doses cause apoptosis

**Figure 3:**
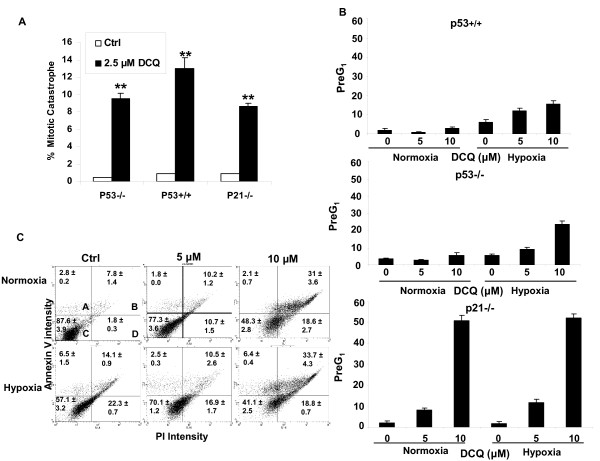
**DCQ induces mitotic catastrophe and apoptosis in HCT116 cells**. **(A) **Low concentrations of DCQ triggered mitotic catastrophe in all HCT116 cell lines. Cells were cultured on coverslips and treated at 50% confluency with 2.5 μM DCQ for 48 hrs after which they were fixed and stained with Hoechst and viewed under a fluorescent microscope using UV. (**) indicates p < 0.001 (one way ANOVA) with respect to the Ctrl. **(B) **Higher concentrations of DCQ (5 and 10 μM) induced increases in the PreG_1 _phase population more so under hypoxia. Treatment with DCQ in normoxia or hypoxia was for 6 hrs, after which cells were harvested immediately and DNA was stained with PI for analysis with FACScan flow cytometry. The percentage of Pre G_1 _cells was calculated using Cell Quest. **(C) **Annexin V assay showing the apoptotic/necrotic response of p53^+/+ ^cells exposed to 5 or 10 μM DCQ for 6 hr in normoxia or hypoxia. Apoptosis was assayed 24 hr after drug treatment, and appeared to be enhanced in hypoxia at higher drug concentrations. Quadrant A = apoptotic cells, B = apoptotic+necrotic, C = normal, D = necrotic. The experiment was repeated twice each in duplicates.

To determine the mode of cell death induced by DCQ, we exposed HCT116 cells to low (2.5 μM) or high (5 or 10 μM) concentrations of DCQ under normoxia or hypoxia and analyzed cells by flow cytometry, Hoechst staining and Annexin V techniques 6-24 hrs later. Depending on the severity of DNA damage, cancer cells have been shown to die by apoptosis, necrosis or mitotic catastrophe. Recent evidence has shown that low doses of anticancer drugs, like paclitaxel, induce mitotic catastrophe followed by apoptosis [27]. In our system, we observed signs of mitotic catastrophe only in response to lower concentrations of DCQ (2.5 μM) for 48 hrs. Under these treatment conditions, the nuclei of all three HCT116 cells became significantly larger and some cells contained several nuclei of unequal sizes, which are characteristic of mitotic catastrophe (Figure 3A). Mitotic catastrophe was not observed in cells exposed to higher concentrations of DCQ (5 or 10 μM) under normoxia or hypoxia (data not shown). The Pre-G_1 _increase is indicative of apoptosis and necrosis as evidenced by the higher percentage of Annexin-positive apoptotic cells (Figure 3C). In Figure 3C, quadrant A represents apoptotic cells, B apoptotic and necrotic cells, C normal cells and D necrotic cells. The percentage of apoptotic and necrotic cells increased from 8% and 14% in control normoxic and hypoxic cells respectively, to 31% and 34% in cells treated with 10 μM DCQ. The apoptotic response and Pre-G_1 _phase increase was 2-5 fold higher under hypoxia than normoxia depending on the cell line, which was in agreement with the clonogenic and MTT assay observations (Figures 1 and 2). Again, the p21^-/- ^cells showed the greatest increase in the Pre-G_1 _population (Figure 3B), further confirming the higher drug sensitivity of this cell line.

### There is no dose-response toxicity by DCQ in normal intestinal cells

**Figure 4:**
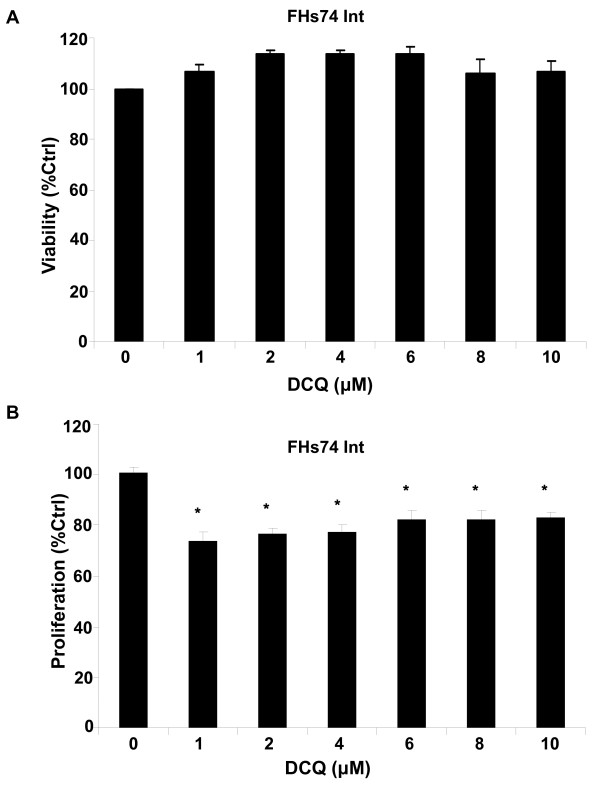
**DCQ is not cytotoxic to normal intestinal cells**. DCQ at concentrations of up to 10 μM did not reduce FHs74 Int human normal intestinal cell viability. At 50% confluency, cells were exposed to DCQ for 6 hr or were left untreated. Viability was assessed by the Cytotox 96 non-radioactive assay **(A) **and by the MTT-based Promega assay **(B)**. Values are averages ± SE of two independent experiments each done in triplicates. The experiment was repeated three times each in triplicates.

To determine if DCQ is an effective anti-tumor drug that specifically targets cancer cells and spares normal ones, we investigated the dose-response toxicity of DCQ in normal human intestinal FHs74 cell line. Treatment of cells with DCQ concentrations of up to 10 μM for 6 hrs was followed by measuring LDH release and cell viability by the MTT-based assay. DCQ was not cytotoxic to the normal intestinal cells (Figure 4A), and although cell viability was reduced by 1 μM of the drug, it did not seem to change much with dose increase (Figure 4B).

### DCQ induces DNA damage and activates ATM in p53^+/+ ^cells

**Figure 5:**
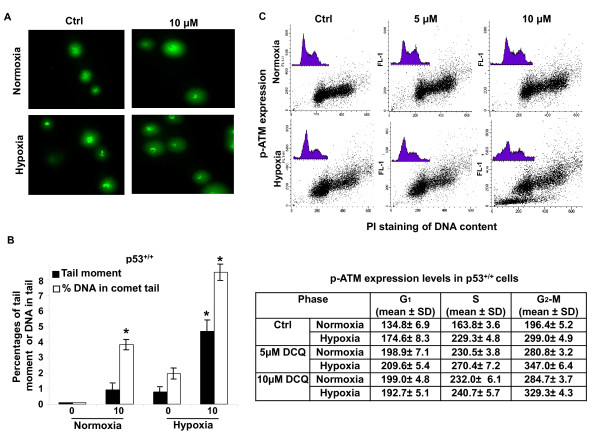
**DCQ induces DNA damage and increases ATM expression in p53^+/+ ^HCT116 cells**. SSB and DSB induced by DCQ in p53^+/+ ^cell line. **(A) **Examples of comets induced by DCQ in cells subjected to the alkaline comet assay. Cells treated with DCQ for 6 hrs in normoxia or hypoxia were collected directly after treatment, subjected to the alkaline comet assay and images were taken using a fluorescent microscope at 40× (oil immersion) magnification. The comets observed by each treatment are directly proportional to the amount of SSBs induced. **(B) **The mean of the parameters (% DNA in comet' s tail and tail moment) are shown in the graphs above. More than 50 cells per treatment were photographed and quantified using TriTek CometScore software. (*) indicates p < 0.05 (one way ANOVA) with respect to control. **(C) **DCQ-induced phosphorylation of ATM in p53^+/+ ^cells at 6 hrs as an indication of DSB. After treatment, cells were fixed and subjected to immunocytochemical detection of ATM phosphorylated on Ser-1981, and stained with PI to detect at the same time p-ATM in each phase of the cell cycle. The mean of the FL-1 intensity ± SD (reflecting the level of p-ATM expression) at the G_1_, S and G_2_M phases of the cell cycle are shown in the table. The experiment was repeated twice each in duplicates.

Next we investigated whether DCQ causes cell death in human colon cancer cells by inducing DNA damage and activating ATM, as similar effects have been observed in EMT6 mouse mammary carcinoma cell lines [11]. For this, we used the p53^+/+ ^cells as model, since this cell line harbors functional p53 and DCQ significantly decreased the induction of HIF-1α by hypoxia in p53^+/+ ^cells (Figure 2B). Cells were treated with DCQ and exposed to normoxia or hypoxia for 6 hrs after which they were subjected to the alkaline comet assay for determining SSB formation and to immunocytochemistry for measuring the extent of ATM activation (an indication of DSB). The extent of SSB formation in response to DCQ was evaluated and quantified using TriTek CometScore, software which calculates different parameters by assuming that the amount of DNA at a certain location (or the intensity of the DNA stain) is proportional to the pixel intensity at that position. Different parameters were used to quantify the extent of DNA damage induced by DCQ including % DNA in comet's tail (representing damaged DNA migrated away from nucleus), and tail moment (% DNA in comet's tail multiplied by the tail length). Under normoxia, DCQ induced a significant increase in the level of SSBs (Figures 5A, B), however, under hypoxia, SSB were augmented by the drug. The tail moment and % DNA in tail moment increased significantly (p < 0.05) in comparison with that of untreated cells (Figure 5B).

Upon DNA damage, cell cycle checkpoints are activated. These DNA repair processes are mediated via two protein kinase pathways: the ATM through Chk2 and ATR via Chk1 [28-30]. ATM, a member of the PIKK family, is mainly activated upon DSB formation by the autophosphorylation of the Ser-1981 [30]. Our results indicated that control cells have basal levels of p-ATM expression which are higher in the G_2_-M population due to the critical role of ATM in mitosis. Exposure of p53^+/+ ^cells to 5 or 10 μM DCQ triggered the activation of ATM by its phosphorylation at Serine 1981 in all the phases of cell cycle and this activation was more pronounced under hypoxia (Figure 5C). Hypoxia alone increased ATM expression, however, the combination of DCQ and hypoxia treatment induced higher levels of p-ATM expression in the G_2_-M phase in comparison with control cells (Figure 5C). These results confirm that DCQ induces DSBs in human colon cancer cells.

### DCQ modulates protein expression of downstream ATM effectors

**Figure 6:**
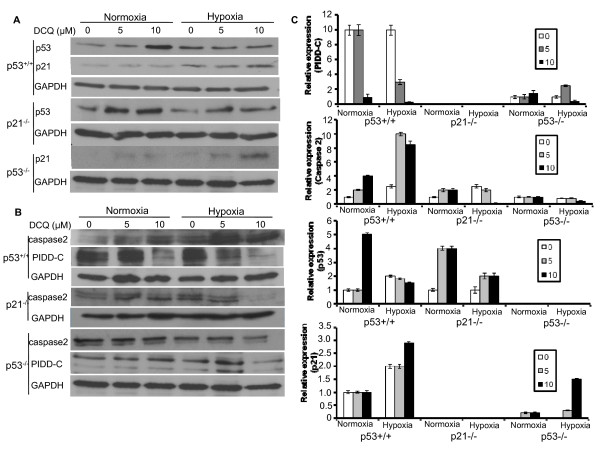
**DCQ modulates the protein expression levels of key mediators of apoptosis and mitotic catastrophe**. At 50% confluency, cells were treated with 5 or 10 μM DCQ for 6 hrs. Whole cell lysates were then immunoblotted with the different primary antibodies and probed with GAPDH to ensure equal loading. **(A) **p53 and p21 protein expression and **(B) **caspase-2 and PIDD-C protein expression in HCT116 cell lines in response to DCQ treatment under normoxic or hypoxic conditions. **(C) **Relative densitometry values of analyzed proteins are plotted. All values were normalized to GAPDH and calculated relative to the control cells cultured under normoxia. The experiment was repeated twice each in duplicates.

Upon DNA damage, one of the important transcription factors activated by ATM through phosphorylation is p53, the activation of which triggers G_1 _or G_2 _arrest (in case of p21 increase) or apoptosis [29,30]. In addition, ATM can lead to the activation of PIDD, an important target gene in a signaling pathway that is initiated by p53, subsequently causing either activation of NFκB-dependent cell survival through PIDD-C or apoptosis through PIDD-CC [31,32]. To assess the effect of DCQ on downstream targets of ATM, we investigated its ability to induce changes in the expression levels of p53, p21, PIDD-C and caspase-2 proteins. Cells were exposed to 5 or 10 μM DCQ and protein changes were monitored 6 hrs post-treatment under normoxia or hypoxia. The p53 protein increased in response to DCQ in all three cell lines except in p53^+/+ ^cells exposed to hypoxia (Figure 6A); p21 protein also increased in all cell lines except in p53^+/+ ^cells exposed to normoxia (Figure 6A). Exposure of the p53^+/+ ^cells to 5 or 10 μM DCQ gradually increased the level of caspase-2, and the upregulation was 8-10 fold higher under hypoxia (Figure 6C). In p21^-/- ^cells, DCQ treatment under normoxia increased caspase-2 expression levels. The exposure of p21^-/- ^cells to hypoxia alone increased caspase-2 expression, however the combination of DCQ and hypoxia reduced it (Figure 6C). DCQ had no effect on caspase-2 protein expression in p53^-/- ^cells which is not surprising, since p53 is known to regulate caspase-2 [33]. Although no direct interaction between p53 and caspase-2 has been observed, it is believed that a functional connection between these two proteins is essential for the initiation of drug-induced apoptosis [34]. Enforced PIDD expression or the over expression of p53 have been shown to promote cell death through the activation of caspase-2 [33,34]. In p53^+/+ ^and p53^-/- ^cells, DCQ downregulated PIDD-C protein expression under normoxia and hypoxia (Figure 6B, C). PIDD-C was not detected in p21^-/- ^cells.

## Discussion

The low oxygen tension in solid tumors is one major factor for tumor resistance to radiotherapy and chemotherapy; therefore there is interest in the discovery of novel drugs that can specifically target tumor cells. In this study, we showed that DCQ is a DNA damaging and apoptotic agent that reduces the viability and colony forming ability of colon cancer cells and is non-toxic to normal intestinal cells. We have shown previously that DCQ is not toxic to normal mouse intestinal Mode K and IEC-6 cells [9] or to normal mouse mammary SCP2 cells [unpublished findings], suggesting the selectivity of this drug to cancer cells.

The reduction of viability and colony survival by DCQ was more pronounced under hypoxia than normoxia and was evident in all HCT116 cell lines, particularly in p21^-/- ^cells which showed greater drug-induced increases in Pre-G_1_. The apoptotic effects of DCQ in p53^+/+ ^cells correlated with an increase in the pro-apoptotic caspase-2 protein, inhibition of the pro-survival protein PIDD-C, and increase in p-ATM expression, a major protein kinase involved in repair of DSB.

DCQ belongs to a group of heterocyclic compounds with potent hypoxic cytotoxic activities [4], of which the heterocyclic di-N-oxide TPZ is in phase III clinical trials [35]. The hypoxia toxicity of TPZ is due to the production of radicals that form strand breaks in the DNA [35,36]. Under normoxic conditions, the radical is back-oxidized to the nontoxic original compound with the related production of the much less toxic superoxide radical [36]. Unlike TPZ which is active only under hypoxia, DCQ appears to be equally active in HCT116 cells cultured in both normoxic and hypoxic environments which explains the low HCR ratios of (< 1.5) specific for this cell line. This is in contrast to the high HCR ratios (> 100) in T-84 human colon cancer cells [4], suggesting that the hypoxia potency of DCQ is cell-type specific.

Hypoxia-Inducible Factor-1alpha (HIF-1α) is an important cellular transcription factor that is stabilized under hypoxia [reviewed in [37]]. HIF-1α regulates the metabolic adaptation to O_2 _deprivation in tumors, and plays an essential role in allowing tumors to escape apoptotic mechanisms and becoming angiogenic [37,38]. DCQ has previously been shown to decrease HIF-1α mRNA and protein expression levels in mouse mammary carcinoma cell lines [8]. Here we show that DCQ decreased HIF-1α protein expression in p53^+/+ ^and p53^-/- ^HCT116 cell lines, despite the constitutive expression of HIF-1α under both normoxic and hypoxic conditions in the latter cell line (Figure 3B). This result is interesting in light of the literature showing that hypoxia selects for p53 mutant tumors [26]. Furthermore, interfering with HIF-1α is important for effective antitumor therapy [37].

Mitotic catastrophe occurs during, or shortly after, dysregulated or failed mitosis, and is believed to be fundamentally different from apoptosis. Despite its distinctive morphology, mitotic catastrophe may represent a pre-stage of apoptosis [17]. Apoptosis, however, is not always required for the lethal effect of mitotic catastrophe, since abnormal mitosis can lead to cell death through apoptosis and necrosis based on the molecular profile of cells [17]. In our system, we show that at low doses of DCQ (2.5 μM), the three HCT116 cell lines displayed an entirely different nuclear morphology with enlarged nuclei, a morphology that has been previously known to result from mitotic catastrophe (Figure 3A). No signs of mitotic catastrophe were observed at higher DCQ concentrations of 5 or 10 μM or at shorter incubation times (data not shown). Rather high drug concentrations induced a significant increase in Pre-G_1_, a sign apoptosis and necrosis (Figure 3B).

Having determined that DCQ induces mitotic catastrophe at lower drug concentrations and apoptosis at higher concentrations, we next investigated whether it causes DNA damage and activates ATM in the p53^+/+ ^cell line. DNA damage imposes a threat to the survival of cells if the damage is unrepaired [12]. As a response to the damage, cells activate the DNA damage checkpoint. DSBs are detected by two main players in the DNA damage checkpoint: ATM and DNA-PK. Signal transduction, induced by the activation of ATM, can cause cell-cycle arrest, repair, and cell death. ATM plays a critical role in S and G_2_-M phase arrest. Activated by DSBs, ATM becomes phosphorylated at Ser-1981 [30,39]. Our experiments using the alkaline comet assay show that DCQ causes SSB and DSB in p53^+/+ ^cells under normoxic and hypoxic conditions, however, the extent of DNA strand breaks was higher under hypoxia. Interestingly, ATM was activated in all phases of the cell cycle in response to the DNA damage induced by DCQ especially under hypoxia (Figure 5), suggesting a positive correlation between the extent of DNA damage and the activation of ATM.

The p21 gene is transcriptionally activated by p53 and is responsible for the p53-dependent checkpoint which induces cell cycle arrest after DNA damage. Enforced p21 expression is known to result in a consistent, but partial, protection of cells from apoptosis [40,41]. In HCT116, a significant increase was observed in p21 expression in response to DCQ treatment under hypoxia both in p53^+/+ ^and p53^-/- ^cells suggesting that p21 activation is independent of p53. In addition, the decrease in the expression levels of the prosurvival PIDD-C protein coupled with the increase in proapoptotic caspase-2 in p53^+/+ ^cells, appears to have committed the cells to apoptosis. In p53^-/- ^and p21^-/- ^HCT116, the apoptotic cell death occurred independent of caspase-2 activation and/or PIDD-C downregulation (Figure 6B), suggesting the involvement of other mediators of apoptosis.

It has been debated if mitotic catastrophe results in cell death via caspase2-dependent or - independent mechanisms [42]. At least three lines of evidence indicate that, in our cell system, mitotic catastrophe is independent of p53 and/or caspase-2 activation. First, mitotic catastrophe occurred in drug treated cells that are null for p53 (Figure 3A). Second, in p53^+/+ ^cells, higher doses of 5 and 10 μM DCQ induced caspase-2 activation (Figure 6B), while morphological changes of mitotic catastrophe were observed at lower drug doses (Figure 3A). Third, the three HCT116 cell lines displayed signs of mitotic catastrophe, yet only p53^+/+ ^cells showed activation of caspase-2, and especially under hypoxia (Figure 6B). Although, it was suggested that the presence of functional p53 in cancer cells enhanced their sensitivity to hypoxia, DCQ-induced apoptosis in HCT116 was not dependent on the presence of the p53 gene, as the Pre-G_1 _increase was evident even in cells lacking the p53 gene (Figure 3B).

## Conclusions

DCQ is a selective cytotoxin in HCT116 human colon cancer cells and its toxicity is independent of p53 and p21. DCQ toxicity is associated with enhanced DNA damage, activation of the ATM damage repair pathway, as well as induction of apoptosis or mitotic catastrophe depending on the drug concentration used. The absence of major toxicity to normal cell lines (human intestinal cells in this study and mouse intestinal cells and mouse mammary cells in previous studies) makes DCQ an interesting compound with potential anticancer activities against colon cancer, and therefore a drug for further testing.

## Competing interests

The authors declare that they have no competing interests.

## Authors' contributions

ME carried out the experiments in the study and prepared the figures. FG was involved in revising the manuscript. MH provided the compound and revised the manuscript. HGM conceived of the study, designed the experiments and drafted the manuscript. All authors read and approved the final manuscript.
